# Clinical characteristics and surgical outcomes of isolated inferior rectus palsy

**DOI:** 10.1186/s12886-021-02121-z

**Published:** 2021-12-07

**Authors:** Licheng Fu, Binbin Zhu, Jianhua Yan

**Affiliations:** State Key Laboratory of Ophthalmology, Zhongshan Ophthalmic Center, Sun Yat-sen University, 54 Xianlie Nan Road, Guangzhou, 510080 Guangdong Province China

**Keywords:** Inferior rectus muscle, Paralytic strabismus, Dysplasia, Extraocular muscle, Strabismus surgery

## Abstract

**Aim:**

As isolated inferior rectus muscle (IRM) palsy represents a rare clinical entity, very limited information is available on this condition. The aim of this report was to elucidate the etiology, clinical characteristics and surgical outcomes of isolated IRM palsy.

**Methods:**

Isolated IRM palsy cases who underwent surgical treatments at the Zhongshan Ophthalmic Center, Sun Yat-sen University, China over the period from January 2008 to June 2019 were reviewed retrospectively. Data evaluated from these cases included their etiology, ocular alignment, ocular motility, surgical procedures and surgical outcomes.

**Results:**

A total of 61 patients (40 males, 21 females) were included in this review. Their mean ± SD age was 27.21 ± 16.03 years (range: 2 to 73 years). In these cases, 32 (52.5%) involved traumatic injury, 28 (45.9%) congenital hypoplasia or absence of inferior rectus and 1 (1.6%) with thyroid ophthalmopathy. The right eye was affected in 33 patients (54.1%), the left in 24 patients (39.3%), and both eyes in 4 patients (6.6%). The main clinical presentations consisted of hypertropia of the affected eye, motility limitation in abduction and depression and incyclotropia. After treatment consisting of various surgical approaches, including muscle repair or resection of the affected inferior rectus, recession of ipsilateral superior rectus, elongation of contralateral superior oblique and partial transposition of the horizontal rectus, the isolated IRM palsy was rectified in 49 patients (80.4%) with one surgery, while 11 cases (18.0%) required two surgeries and 1 case (1.6%) needed three surgeries. Finally, 52 patients (85.2%) showed a complete recovery, 6 (9.9%) improved and 3 (4.9%) experienced a surgical failure.

**Conclusion:**

The main etiologies of isolated IRM palsy involved traumatic injury and developmental events. Overall, surgical outcomes of the various approaches employed were quite effective.

## Introduction

Isolated inferior rectus muscle (IRM) palsy without involvement of other muscles supplied by the oculomotor nerve is very rare [1–4], with only a few reports in the literature describing this condition. Von Noorden et al. [1] reported that its etiologies included conditions such as trauma, congenital, idiopathic and vascular events as well as myasthenia gravis. Overall, they found that favorable surgical outcomes were obtained in all 21 cases. After an analysis of 28 patients with this condition, Awadein et al. [2] concluded that it was difficult to determine a definitive etiology as based solely on clinical features and orbital imaging. Choi et al. [3] reported that of the 44 cases of acquired IRM paresis, vascular, traumatic and inflammatory events were the main causes, and 95% of these cases showed a complete recovery with or without any treatment. However, Akbari et al. [4] concluded that a considerable number of IRM palsy cases could be successfully treated with IRM resection.

From this limited review involving IRM cases, it is clear that pertinent clinical characteristics and surgical management of congenital and acquired IRM palsy are sorely lacking. Protocols for the treatment of this condition as well as its etiology need to be established. Such informations will be required to resolve some of the clinical challenges with regard to the selection and implementation of appropriate surgical corrections of isolated IRM palsy after failure of conservative treatments. In this report, we review 61 cases of IRM palsy who underwent strabismus surgery, which represents the largest case sample of this condition within the literature. Unlike that of previous reports, we found that an etiological diagnosis of IRM palsy could be achieved in all patients after conducting a detailed clinical and orbital imaging evaluation. In addition, ipsilateral IR resection with/without superior rectus (SR) recession produced satisfactory surgical outcomes in a large percent of these cases.

## Patients and methods

A retrospective analysis of patients with isolated IRM palsy who underwent surgical treatments at the Zhongshan Ophthalmic Center of Sun Yat-sen University, China from January 2008 to June 2019 was conducted. Written informed consent to participate in this study was obtained from all patients. Institutional approval for this study was obtained from the Research Ethics Board of the Zhongshan Ophthalmic Center (Approval No:2019KYPJ103), of Sun Yat-sen University, China, and all procedures were performed in accordance with the 1964 Declaration of Helsinki. All patients were seen and surgically treated by the corresponding author (JH Yan). The diagnosis of IRM palsy was according to previously established [1–4] criteria and included: (1) Significant hypertropia of the affected eye with maximal deviation in the field of IRM action as indicated by the prism and alternate cover tests in nine diagnostic positions, (2) Patients with acquired IRM palsy experiencing vertical diplopia that was also aggravated in abduction and depression, (3) On ductions and versions, maximal deficiencies in ocular motility were in abduction and depression of the involved eye, (4) Incyclotropia as detected by either objective or subjective tests, (5) Patients with congenital and traumatic IRM paresis had been confirmed by orbital computed tomography (CT) and/or magnetic resonance imaging (MRI), (6) A negative result in the forced duction test as performed in all patients while under general anesthesia and (7) Patients with or without A-pattern. The exclusion criteria included: (1) Patients who did not receive strabismus surgery, (2) Patients who could not be followed up for at least 6 months and (3) An angle of deviation in patients with acquired IRM paralysis that was not stable for 6 months, (4) Patients who had surgery history of strabismus correction.

The following clinical characteristics were recorded from the patients’ charts: age, sex, occupation, affected eye, family history, age at onset, age at surgery, the best corrected visual acuity, cycloplegic refraction, anterior segment, fundus photograph, ocular motility, ocular alignment at distance (6 m) and near (33 cm) by prism and alternative cover tests in nine diagnostic positions, stereoacuity at distance and near, presence of an A or V pattern, and surgical methods employed. A special headwear device was used to quantitatively evaluate compensatory head posture. The amount of cyclotropia was measured by fundus photograph (obtained by recording the fovea position as related to the optic disc) and/or with use of double Maddox rod testing. Limitations in eye movement in all diagnostic gaze of IRM were measured using a 5-point scale (0 to − 4) as described previously [2, 4]. Orbital MRI and/or CT examinations were applied to patients with a history of trauma and congenital hypoplasia of extraocular muscles.

All surgeries were performed through fornix conjunctival incisions while patients were under general anesthesia. The forced duction test was routinely performed during surgery. If the IRM retained residual function and hypertropia in the primary position was < 15 PD, only ipsilateral IR resection or ipsilateral SR recession were performed. With a hypertropia in primary position between 15 and 30 PD, ipsilateral SR recession with/without IR resection was performed. If the IRM showed complete paralysis or in cases with congenital absence of IRM, a combination of ipsilateral SR recession and partial (1/2) horizontal rectus muscle transposition were performed. In some cases, we employed an anterior transposition and resection of the ipsilateral inferior oblique muscle to correct the hypertropia. If the contralateral superior oblique muscle was obviously overacted and the vertical deviation was < 15 PD, a contralateral superior oblique lengthening surgery was selected. Recession and resection of the horizontal rectus were performed in cases where there was a simultaneous esotropia or exotropia. In patients with a previous history of retinal detachment surgery or muscular injury, surgical techniques involving scar release and IRM repair were used. Patients were followed up at 1 day, 1 week, 2 months, 6 months, 1 year and 2 years after surgery.

A complete cure was defined as a vertical deviation of ≤3 PD in primary and reading positions, an absence of diplopia and normal head posture. An improvement was considered to be present when the vertical deviation was > 3 PD but ≤10 PD in primary position with an anomalous head posture. Failures were defined as > 10 PD vertical deviation and persistent diplopia in the primary position or downward gaze [1].

Statistical analysis was performed with use of the SPSS version 23.0 (SPSS Inc., Chicago, IL). All values presented represent means±SD. Pre- versus post-operative values were compared using a paired t-test. A *p*-value < 0.05 was required for results to be considered statistically significant.

## Results

### Etiologies

A total of 61 patients (40 males, 21 females) were included in this review, with their mean ± SD age being 27.21 ± 16.03 years (range: 2 to 73 years). The right eye was affected in 33 patients (54.1%), the left in 24 (39.3%) and both eyes in 4 patients (6.6%). The underlying causes of the isolated IRM palsy included trauma (*n* = 32, 52.5%), congenital IRM hypoplasia (*n* = 28, 45.9%) and thyroid ophthalmopathy (*n* = 1, 1.6%) (Table 1). In the trauma group, 22 patients (*n* = 22, 36.1%,) experienced a traumatic rupture of the IRM, 7 patients (11.5%, 7/61) an orbital blow-out fracture. In the 3 cases of iatrogenic injury (4.9%, 3/61), 1 case involved complications from endoscopic sinus surgery, 1 case from scleral buckling for retinal detachment and 1 case due to effects resulting from intracranial tumor resection. In the congenital group, 20 patients (32.7%) showed a congenital dysplasia of the IRM (19 unilateral and 1 bilateral), 6 cases (9.9%) a congenital absence of the IRM (5 unilateral and 1 bilateral) and 2 cases (3.3%) involved a co-existent congenital hypoplasia and absence of IRM. The patient with thyroid ophthalmopathy was somewhat unique, presenting with typical IRM palsy, negative forced duction test, a normal size of their extraocular muscles and a definitive exclusion of myasthenia gravis.

**Table 1:** Etiologies. Clinical findings. Surgical procedures of Isolated IR Palsy (*N* = 61)

Eologies	N	Clinical Findings	N	Surgical Procedures	N
Traumatic	32	Diplopia	32	IR resection	13
Congenital	28	Abnormal head position	15	SR recession	8
Congenital hypoplasia of IR	20	Limitation of eye movement	61	SR recession and IR resection	18
Congenital absence of IR	6	Intorsion	61	Anastomosis and/or repair of the affected IR	22
Congenital hypoplasia and absence co-exist of IR	2	Esotropia	8	Elongation of contralateral SO or/and the ipsilateral IOAT	4
Thyroid ophthalmopathy	1	Exotropia	9	Horizontal rectus recession and resection	5
		Amblyopia	6	Ipsilateral SR recession and partial tendon transposition of horizontal rectus	4
		Nystagmus	5		
		Refractive error	31		
		Anisometropia	7		

*IR* Inferior rectus, *SO* Superior oblique, *SR* Superior rectus, *IOAT* Inferior oblique anterior transposition, *N* number

### Clinical characteristics

The clinical features of IRM palsy differed between the congenital and traumatic injury groups. The main clinical findings were hypertropia and abnormal head position in the congenital group, and diplopia and hypertropia in the traumatic group. In traumatic group, other complications included other ocular traumas such as blow-out orbital fractures in 7 patients, eyelid retraction (12 patients), laceration of lacrimal canaliculus (3 patients), symblepharon (5 patients), conjunctival laceration (22 patients) and conjunctival granuloma (1 patient) and optic nerve contusion and atrophy (2 patients). Thirty two cases (52.5%) experienced diplopia and 45 (73.8%) showed abnormal head positions. In this latter group, the abnormal head positions mainly included chin-down (100%) and slight rotation toward the IRM involved side (100%) with a head tilt to either the ipsilateral (43%) or contralateral (57%) shoulder. Compensatory head postures were measured with use of a special headwear device in 14 patients (14/45–31.1%). The mean alteration in chin-down positioning was 18 degrees (range: 6.9 to 36.5), the average of ipsilateral face-turn was 10.13 degrees (range: 6.3 to 19.8), while the mean head tilt to the ipsilateral or contralateral shoulder was 8.5 degrees (range: 4.5 to 12.5). Eight patients (8/45–17.8%) showed a difference in vertical misalignment between right and left head tilts, with the mean difference being 8.01 ± 10.06 PD (range: − 12 to 20 PD, *P* = 0.21). The overall mean hypertropia in primary position at distance was 19.62 ± 11.37 PD, with 33 patients (54.1%) showing ranges of 10–25 PD, 24 (39.3%) from 26 to 50 PD and 4 patients (6.6%) showing a range from 51 to 80 PD. Mean hypertropia in primary position at near was 19.15 ± 9.90 PD while the mean preoperative hypertropia in the field of IR muscle action was 27.31 ± 12.65 PD. The mean value of intorsion was 16.06 ± 2.66 D, with intorsion being detected in all patients (100%), among which 14 cases (22.9%) showed clear fundus photography of both eyes. There were 8 cases of coexistent esotropia and 9 of coexistent exotropia (2 cases with A-pattern exotropia). Mean limitations of duction in the gaze of IRM was − 2.52 ± 1.51, with limitations of − 3 to − 4 in 37 cases (60.7%) and − 1 to − 2 in 24 cases (39.3%). Twenty-six cases (42.6%) had fusion function and 10 (16.4%) stereopsis function. Six cases (9.9%) had amblyopia, 5 (8%) nystagmus, 31 (50.8%) refractive error, and 7 cases (11.5%) of anisometropia were observed in our group (Table 1).

Images from orbital CT and/or MRI scans were available in 38 cases (38/61–62.3%), with CT scans alone in 20 cases, MRI alone in 18 cases and both MRI and CT examinations in 16 cases. In patients with IRM trauma, results of the orbital CT/MRI revealed blow-out orbital fractures (7 patients), IRM rupture or disorganization (16 patients) and eyelid swelling (2 patients). In patients with congenital anomalous IRM, orbital MRI/CT scans showed obvious thinning or atrophy of the IRM belly in 16 patients and a complete absence of IRM in 4 patients.

### Surgical methods and outcomes

**Fig. 1:**
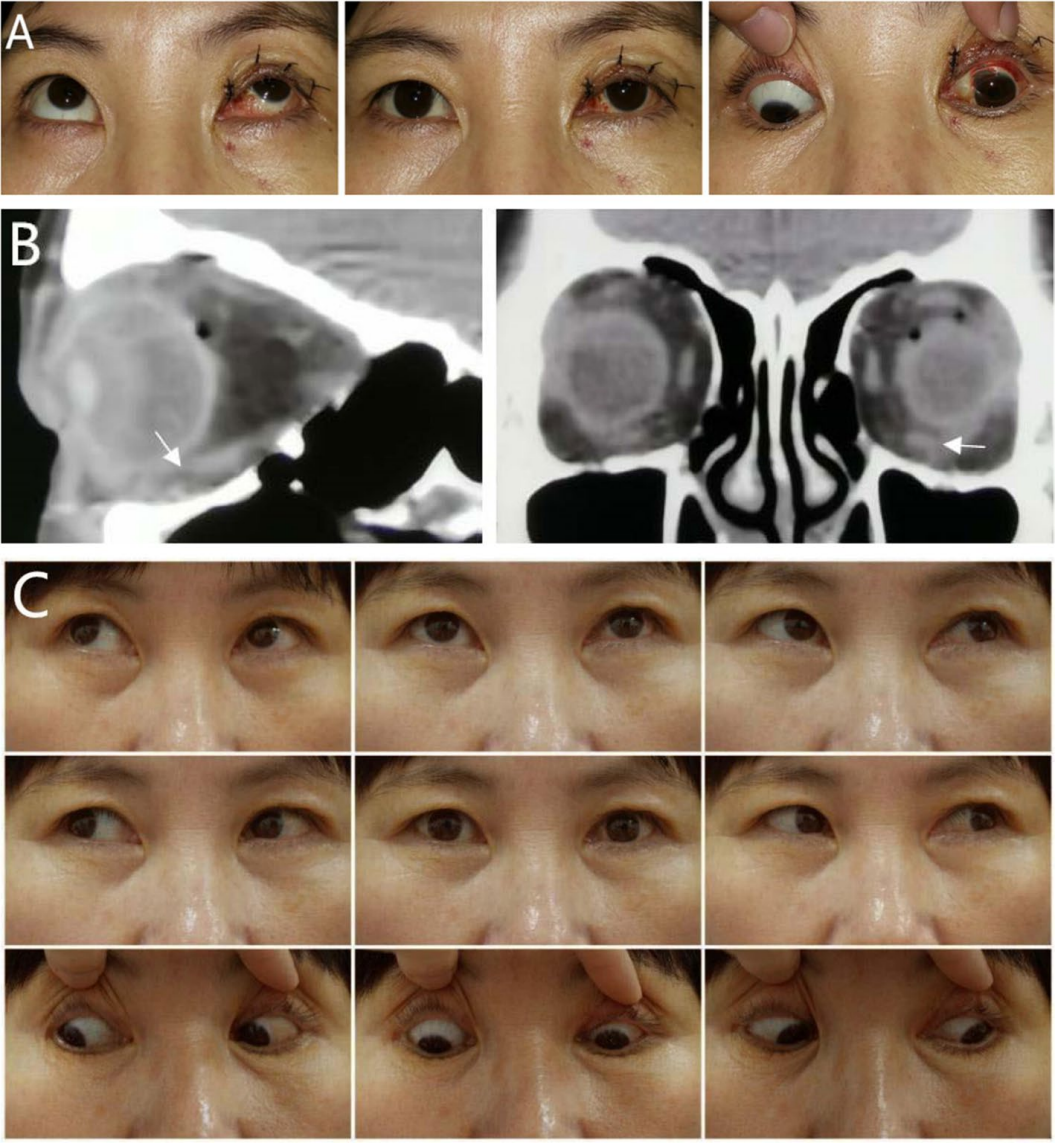
**A-C.** Female, aged 46. Traumatic rupture of inferior rectus muscle (IR) of the left eye by an iron hook. **A**. she had 18 PD left hypertropia in the primary position and − 3 under-action of down gaze. She also had laceration of both conjunctiva and lacrimal canaliculus of the left upper eyelid due to iron hook injury, and had received anastomosis 20 days before strabismus surgery. **B** Orbital CT scans showed that the left inferior rectus just beneath the eyeball was ruptured (the white arrow). **C** Six months after surgical repair of IR combined with 3 mm resection of the IR of the left eye, she showed orthophoia with diplopia free in all directions and normal ocular motility

**Fig. 2:**
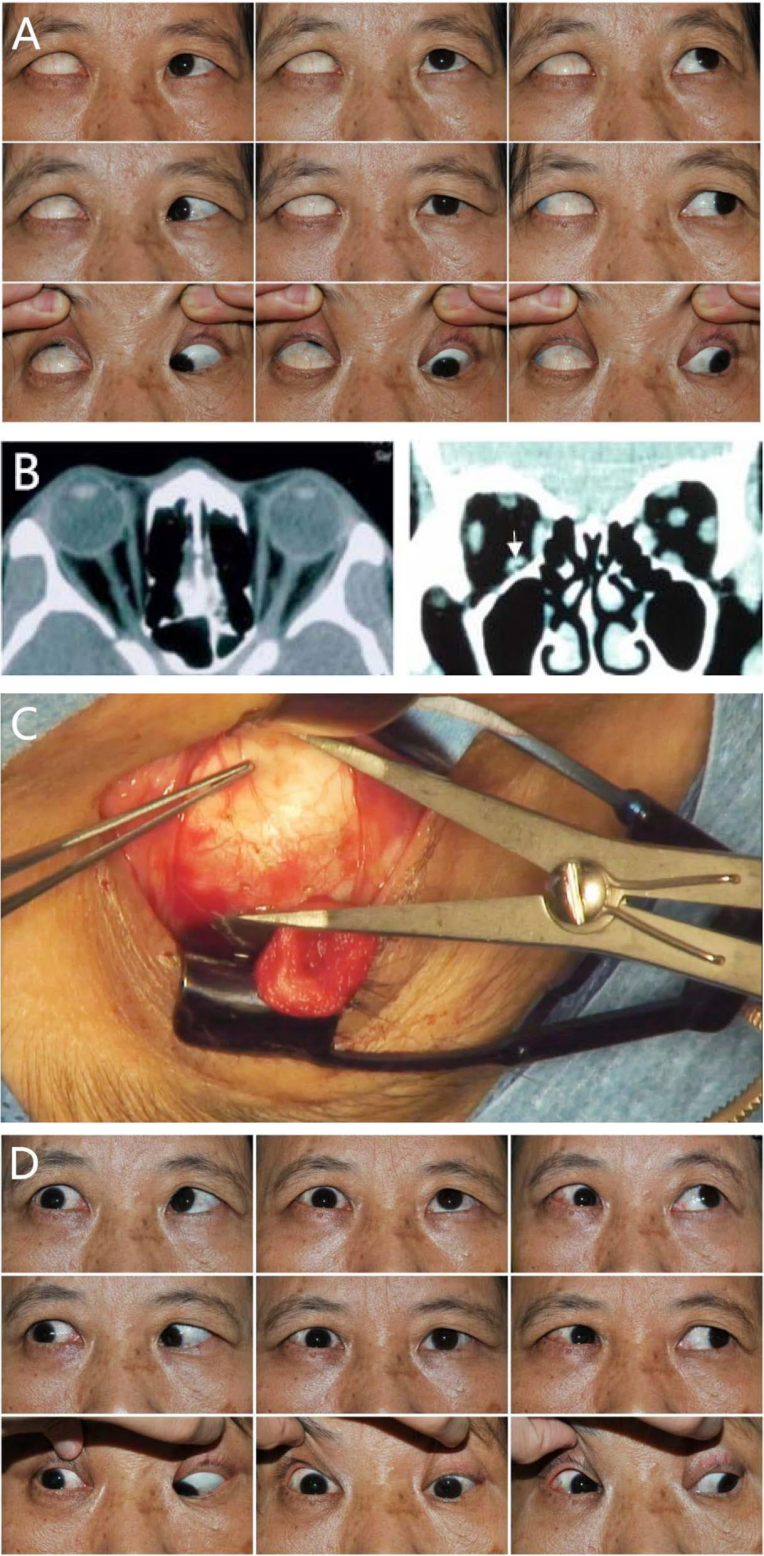
**A-D**: Congenital absence of the right inferior rectus muscle in a 51-year-old female. **A** Clinical 9-gaze photo showing a > 45° hypertropia of the right eye. It was not possible to view the right cornea in the primary position and her right eye was nearly fixed in an extremely high position. **B** Orbital CT scans revealed an absence of a right inferior rectus muscle. However, based upon images from the coronal plane of the CT scan it initially “appeared” that an inferior rectus muscle was present. After careful review of the CT scans, what appeared to be the inferior rectus muscles in the coronal plane was actually the optic nerve which had altered its position due to the extremely high position of the right eye (the white arrow). **C** It was not possible to locate the inferior rectus muscle during the strabismus surgery, however, the anterior ciliary vessels coming from the inferior rectus did exist in this patient. **D** A superior rectus myotomy and anterior transposition of the inferior oblique muscle to the original position of the IR after 8 mm resection of the inferior oblique muscle of right eye was performed. Picture was taken 2 months after the strabismus surgery when she was orthophoria in the primary position, with −2 under-action in downward gaze and − 1 under-action in up gaze motility

A total of 74 surgeries of various types were performed in these 61 cases of IRM palsy (Table 1). In most patients IRM resection or IRM repair combined with or without ipsilateral SR recession were performed (Fig. 1A-C). The amount of IRM resection ranged from 3 to 9.5 mm, while that of SR recession ranged from 3 to 10 mm. Other surgical procedures included contralateral superior oblique weakening and/or anterior transposition of the ipsilateral inferior oblique muscle (Fig. 2A-D) as well as a combination of ipsilateral SR recession and partial transposition of horizontal rectus. Simultaneous horizontal rectus recession and resection were performed in 5 cases. Successful outcomes to a single surgery were achieved in 49 patients (80.4%) (Figs. 1, 2), while 11 cases (18%) required a second surgery and one patient (1.6%) needed three surgeries. Our patients were followed up for an averaged 9.1 ± 1.8 months (range: 6 to 76 months). After surgery, 30 patients (49.2%) had fusion function and 22 (36.1%) stereopsis function. Among the 32 patients with diplopia prior to surgery, 24 showed a complete recovery, 6 improved and only 2 patients failed to show any recovery in response to a subsequent surgery. In the 45 patients with abnormal head positions, normal positions were restored in 39 patients, 3 improved and 3 patients remained unchanged. Mean final postoperative hypertropia in primary position was 4.31 ± 4.17 PD, while 5 cases had mild incyclotropia (< 5 D) and 3 cases had excyclotropia between 2 to 8 D. Mean final postoperative intorsion was 3.64 ± 1.80 D and mean correction of intorsion was 12.41 ± 2.22 D. Limitations of ocular motility in the field of IRM action was restored in 38 cases (62.3%), but remained at a scale of − 3 to − 4 in 11 cases (18.0%) and − 1 to − 2 in 12 cases (19.7%) after strabismus surgery. The final postoperative limitation of ocular motility was − 2.52 ± 1.51 (Table 2).

**Table 2:** Pre- and Postoperative Deviations and Motility in Isolated IR Palsy

	Preoperative (Mean ± SD)	Postoperative (Mean ± SD)	*P*-value
Hypertropia in Primary Position (Far)	19.62 ± 11.37PD	4.73 ± 4.91PD	0.001
Hypertropia in Primary Position (Near)	19.15 ± 9.90PD	4.31 ± 4.17PD	0.001
Hypertropia in the field of IR muscle action	27.31 ± 12.65PD	7.91 ± 5.61PD	0.001
Intorsion(14patients)	16.06 ± 2.66D	3.64 ± 1.80D	0.036

*D* degree, *SD* standard deviation, *PD* Prism Diopter

### Pathological findings

**Fig. 3:**
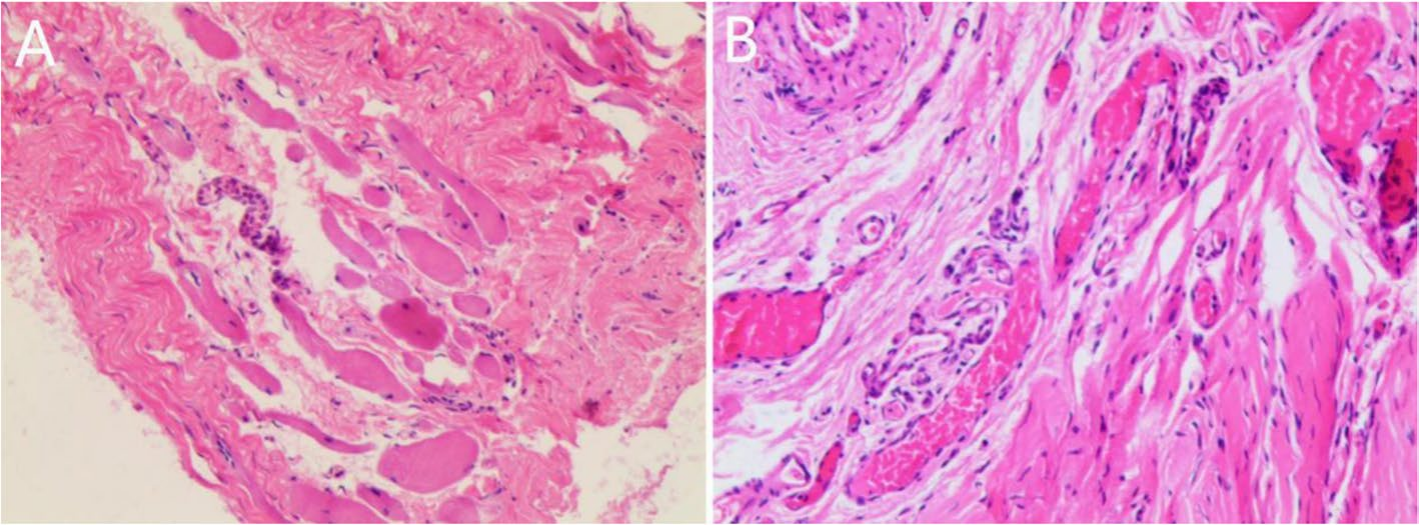
**A-B** Pathological examinations of congenital dysplasia and traumatic injury of inferior rectus muscle. **A** A large amount of denatured fibrous tissue and some undeveloped striated muscle were found in cases with congenital IRM hypoplasia (HE× 40). **B** In cases with IRM injury, some derangement of striated muscle, a moderate amount of proliferated collagen fibrous tissues along with varied sizes of vascular tissues were found in the resected sample (HE × 40)

Pathological examinations of the resected IRM were performed in 31 patients (16 in the congenital group and 15 in the trauma group). Extremely thin and weak IRMs were observed in the 8 cases with congenital hypoplasia. Moreover, a substantial amount of obviously denatured fibrous connective tissue and portions of undeveloped striated muscle were present. In cases with IRM injury, some derangement of striated muscle, along with a moderate amount of proliferated collagen fibrous tissue and varied sizes of vessels were found (Fig. 3A-B).

## Discussion

### Etiologies and clinical finding

With regard to isolated IRM palsy etiology, our findings were particularly intriguing indicating that it appears to result from an approximately equal bases of trauma (52.5%) and congenital dysplasia (45.9%). These findings were similar to those reported by Akbari et al. [4] and von Noorden et al. [1], but differed from that of Choi et al. [3], Spoor TC et al. [5] and Almog Y et al. [6]. Spoor TC et al. [5] found myasthenia gravis presenting as an isolated IRM palsy of acute onset, Almog Y et al. [6] detected myasthenia gravis may affect any of the six extra-ocular muscles, six (20%) isolated inferior rectus was affected in his 30 patients with a clinical diagnosis of myasthenia gravis. A variety of presumed etiologies for isolated IRM palsy had been reported in previous literature, including congenital [1, 4], cerebral infarction (vascular) [3, 4, 7, 8], trauma (especially orbital blow-out fracture) [1–4, 9, 10], infection (brain tuberculosis) [3, 11], effects associated with surgeries for cataracts [12, 13], strabismus [2] and retinal detachment [14], multiple sclerosis [15], midbrain cavernous hemangioma [16], myasthenia gravis [1, 3, 5, 6], thyroidal ophthalmopathy [3] and idiopathic [1]. Variations in patient populations may be responsible for these differences in reported etiologies. For example, patients in the Choi et al. study [3] were from four centers of neurology specialties and two ophthalmology clinics, where higher rates of cardiovascular/cerebrovascular diseases and neurological disorders would be present. Our cases represented patients from a specialized ophthalmic hospital, who often seek specific ophthalmic-related surgical treatments and have with fewer systemic diseases.

The underlying mechanisms of isolated IRM palsy also contribute to the determination of the etiology of this condition. These underlying mechanisms can include either damage to the muscle itself within the orbit (peripheral reason) or damage to the oculomotor nerve branch responsible for IRM function (central reason). Therefore, based upon this dichotomy of underlying mechanisms, peripheral reasons for IRM palsy would include effects such as trauma, congenital hypoplasia or absence of IRM, idiopathic inflammation, myasthenia gravis and thyroid ophthalmopathy, while central reasons would involve the damaged cerebral nuclei of brain stem due to effects such as vascular, multiple sclerosis and cerebral tuberculosis.

We found that middle-aged patients seemed particularly vulnerable to isolated IRM palsy from trauma with the mean ± SD age of our 32 trauma cases being 32.6 ± 2.4 years (range: 8 to 61 years). The IRM is the most susceptible extraocular muscle for injury due to its exposed position around the periorbital area. Vertical diplopia and limitation of downward gaze were the main symptoms in patients with traumatic IRM palsy, who then often adopt a chin down position to decrease their double vision. With regard to cases with congenital hypoplasia or absence of IRM, they often seek treatment for their vertical deviation. Compensatory head positioning and amblyopia were also common in these cases. The case with thyroid ophthalmopathy was quite unique as this patient had a long history of left hypertropia and previous hypertrophic SR. When referred to us, she demonstrated only IRM palsy with no enlargement of any extraocular muscles and a negative forced duction test.

In diagnosing inferior rectus paralysis, von Noorden et al. stressed that the clinician must rely exclusively on testing the ductions and versions and measuring the deviation in the diagnostic position with either eye fixating; while the three step head tilt test and the presence of incyclotropia are of little, if any, help in the diagnosis of IRM palsy [1]. However, in our hands, we found that the presence of incyclotropia was really quite helpful for the diagnosis of IRM palsy, as it enabled us to differentiate between IRM versus ipsilateral superior oblique palsy. In some circumstances patients with superior oblique palsy show a duction limitation that is also observed primarily in the field of IRM action, but they present with excyclotropia, rather than incyclotropia. Overall, the main clinical characteristics of isolated IRM palsy were hypertropia of the affected eye with maximum hypertropia in the field of action of the affected IRM, limitations of duction and version in abduction and depression and incyclotropia in either the affected or normal eye. Notably, the presence of both ipsilateral and contralateral compensatory head postures showed inaccuracies as based upon the Bielschowsky head-tilt test in the diagnosis of IRM palsy [4]. In our patients demonstrating abnormal head position, 43% showed a tilt to the ipsilateral shoulder and 57% to the contralateral shoulder. Therefore, the direction of the head tilt, as determined with the Bielschowsky head tilt test are only of secondary diagnostic value since they may generate paradoxical results [1]. In addition, CT/MRI imagining of the orbit is helpful for the diagnosis of isolated IRM palsy. For example, an orbital floor fracture or traumatic rupture of the IRM can often be confirmed by orbital imaging and congenital dysplasia/absence of IRM can also be detected by imaging as it can reveal a diminished or absent IRM. Finally, a pathological evaluation of the resected IRM was found to be helpful for the etiological diagnosis of IRM palsy. Notably, large amounts of obvious denatured fibrous connective tissue and portions of undeveloped striated muscle were found in cases with congenital hypoplasia. In IRM injury cases, some derangement of striated muscle and a moderate amount of proliferated collagen fibrous tissues were often present.

### Surgical procedures and outcomes

A number of surgical and non-surgical approaches have been employed for the treatment of IRM palsy. These therapies have included conservative observations [3], AChEI drugs [3, 17], cortisol-based drug s[3], as well as treatments with vasodilators, anti-tuberculosis, thyroxine and neurotrophic drugs, which have been shown to be partially effective [3]. Local injections of botox [1, 18] and prism glasses were also effective when initiated in the early stages of IRM paresis where small deviations were present. Strabismus surgery is indicated when non-surgical treatments prove unsuccessful and vertical deviations were relatively large and stable for over 6 months. However, the selection of a reasonable surgical procedure to achieve a favorable outcome for these patients remains a challenge for strabismus surgeons.

In their case series of 21 patients, von Noorden et al. [1] reported that 14 patients were cured, 6 improved and 1 remained unchanged when IR resection, SR recession, IR resection plus SR recession and contralateral superior oblique recession were performed. Recession of the contralateral superior oblique tendon was used when a marked secondary deviation producing incyclotropia of the non-paralyzed eye was present [1]. Akbari et al. [4] stressed the effectiveness of IR resection in the treatment of IRM paresis. If the primary position hypertropia was < 20 PD, they recommended that only ipsilateral IR muscle resection be performed, but in cases of primary position hypertropia > 20 PD or when significant hypertropia was also observed in the field of the superior rectus (SR) muscle, a combination of ipsilateral IR muscle resection and SR muscle recession was suggested [4]. Nishikawa et al. [19] reported that resection and anterior transposition of the inferior oblique muscle was useful for hypoplasia of an inferior rectus muscle that was accompanied by horizontal strabismus. Paysse et al. [20] advocated that full tendon rectus muscle transposition surgery augmented with posterior fixation sutures and the vessel-sparing three fourths partial tendon transposition modification of this technique are effective for the treatment of a variety of complex vertical and horizontal paralytic ocular motility disorders. Our surgical procedures were consistent with that of von Noorden et al. [1]. When the affected IRM retained residual function, ipsilateral IRM resection with/without IRM resection was performed. In cases with congenital absence or complete IRM palsy, ipsilateral SR recession and partial horizontal rectus muscle transposition were performed. If the contralateral superior oblique muscle was obviously overacted and the vertical deviation was < 15 PD, weakening of contralateral superior oblique was selected. When the IRM was absent, anterior transposition and resection of the ipsilateral inferior oblique muscle was very effective in correcting the hypertropia (Fig. 1). In patients with muscle rupture, surgical techniques of IRM repair were used. In order to avoid anterior segment ischemia, the vertical strabismus should first be corrected in patients with co-existing large horizontal and vertical deviations, the horizontal strabismus could then be corrected with reoperation as performed 3–6 months later. In the cases of our study, patients with IRM palsy responded well to surgical correction. After an average 9.1 ± 1.8 months (range: 6 to 76 months) of follow-up,49/61 patients (80.4%) showed a successful outcome after only one surgery, while 11 patients (18%) required two surgeries and 1(1.6%) had three surgeries. Diplopia had been eliminated or improved in 30 patients but unchanged in 2 patients. Abnormal head positioning was resolved or significantly improved in 42 patients but failed in 3 cases.

It should be noted that our results differ from some of those as reported in the previous literature. First, the extent of surgical IRM resection ranged from 3 to 9.5 mm and SRM recession from 3 to 10 mm. Therefore, when the hypertropia in primary position was between 25 and 45 PD and the IRM maintained partial function, both IR resection or SR recession alone and SR recession combined with IR resection could achieve good results. In most of the cases as reported by von Noorden et al., the amount of recession or resection ranged between 3 and 5 mm (mean = 4 mm), but 2 patients required as much as 6–8 mm resection or recession [1]. However, Akbari et al. stressed that the amount of IR muscle resection must be limited to < 4.5 mm to prevent complications such as limitations in elevation and palpebral fissure narrowing [4]. Second, as the modified surgical amount of IR resection and SR resection were relatively large, the reoperation rate was relatively low in our cases, with 80.4% patients showing a favorable outcome after a single surgery. Our reoperation rate (19.6%) was slightly higher than that of Akbari et al. (13.6%) [4] and slightly lower than that of the von Noorden et al. (24%) [1]. No surgical complications, such as anterior segment ischemia and overcorrection of incyclotropia, were observed in any of the cases when simultaneous IR resection and SR recession were performed in the affected eye. Finally, with the inclusion of a pathological examination of the resected IRM it was possible to confirm the diagnosis of congenital IRM dysplasia in patients presenting with structural changes resulting from traumatic IRM injury.

There are limitations of this study, one of which being its retrospective nature. In addition, all cases were acquired from a specialized eye hospital and all received strabismus surgery. In this way, the limited number of patients with general diseases, such as myasthenia gravis and cerebral vascular lesions, may skew the determinations of the etiologies and thus introduce a sample bias in our case series. Finally, longer follow-up times and larger sample sizes will be needed in future studies.

In conclusion, the main etiologies of isolated IRM palsy involved trauma and congenital dysplasia. Surgical outcomes were satisfactory with a success rate of 85.2%. The extent of the surgery for SR recession and IR resection as applied for large angles of deviation can be increased as needed, with a maximal resection of IR being 9.5 mm and recession of SR being 10 mm.

## Data Availability

The datasets used and/or analysed during the current study are available from the corresponding author on reasonable request.
